# Leaf Trait Differentiations Depend on Plant Size in a Modular Assembled Community of Evergreen and Deciduous Trees From a Degraded Karst Forest Ecosystem

**DOI:** 10.1002/ece3.73996

**Published:** 2026-07-08

**Authors:** Mingjuan Yu, Min Jiao, Xihong Yang, Xiaodong Li, Yuejun He

**Affiliations:** ^1^ Forestry College, Research Center of Forest Ecology Guizhou University Guiyang P. R. China

**Keywords:** leaf functional traits, leaf habit, plant size, strategy differentiation, subcommunity

## Abstract

Generally, a forest community can be viewed as functionally integrated assemblages composed of modular subcommunities representing different functional groups, such as evergreen and deciduous plants, which exhibit distinct trait strategies along the leaf economics spectrum. However, how plant size drives leaf trait differentiation between evergreen and deciduous subcommunities remains unclear. In this study, we surveyed 53 forest plots in a typical subtropical karst forest and measured leaf traits, including specific leaf area (SLA), leaf tissue density (LTD), leaf dry matter content (LDMC), and leaf carbon (LCC), nitrogen (LNC), and phosphorus (LPC) contents, as well as soil physicochemical properties. All woody species were classified into evergreen and deciduous subcommunities based on leaf habit. Relationships among plant size, leaf traits, and soil variables were analyzed using linear regression, principal component analysis, and multiple regression. The results showed that the deciduous subcommunity exhibited significantly greater tree DBH, height, SLA, LDMC, LNC, LPC, and leaf N/P ratio, but lower LCC, C/N, and C/P than the evergreen subcommunity, indicating clear differentiation in leaf trait strategies between the two subcommunities. Additionally, distinct trait responses to increasing plant size in both subcommunities, particularly for SLA, LNC, and C/N, suggest plant size‐dependent trait differentiation. Moreover, the relative effects indicated that evergreen leaf traits were mainly influenced by soil total phosphorus and habitat heterogeneity, whereas deciduous leaf traits were primarily driven by plant size. Overall, our findings demonstrate that modular subcommunities exhibit distinct leaf trait characteristics and resource‐use strategies, with an acquisitive strategy in the deciduous subcommunity and a conservative strategy in the evergreen subcommunity, both closely associated with plant size in degraded karst forests. These results provide new insights into trait‐based community assembly by highlighting size‐dependent differentiation in modular subcommunities, with implications for understanding vegetation functional evolution and informing ecological restoration of degraded karst forest ecosystems.

## Graphical abstract

In degraded karst forests, evergreen and deciduous subcommunities adopt contrasting resource‐use strategies. We surveyed 53 plots and found that plant size drives differentiation in leaf functional traits between subcommunities. These results highlight size‐dependent variation in leaf traits that shape adaptation to environmental conditions.

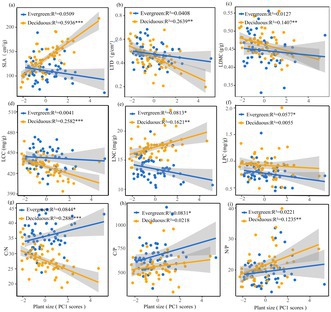

## 1 Introduction

A forest community can be viewed as a functionally integrated assemblage of multiple plant species (Bo et al. 2016; Margalef 1969). Species sharing similar phenotypic and physiological traits often exhibit comparable ecological strategies and can therefore be grouped into functional groups (Bär Lamas et al. 2016). Such groups may be regarded as functional modules that contribute differentially to community organization and ecosystem functioning (Holt and Lawton 1994). Accordingly, these functional modules can be considered subcommunities, comprising organisms that share similar structural and functional characteristics (Ives et al. 2010; Milke et al. 2023). Within forest communities, plants frequently exhibit different habits. For example, natural mixed forests may be structured into distinct leaf‐habit modules, such as evergreen and deciduous species, which differ in their resource‐use strategies (Wang et al. 2023) and exhibit contrasting patterns of leaf morphology and nutrient traits (Conti et al. 2023; Ye et al. 2022). Consequently, evergreen and deciduous species assemblages can be regarded as two modular subcommunities distinguished by different leaf‐habit strategies. Moreover, the leaf economic spectrum (LES) describes coordinated variation among leaf functional traits that reflect resource‐use strategies and life‐history trade‐offs (Wright et al. 2004), thereby influencing plant growth, reproduction, and survival (Violle et al. 2007). Price et al. (2014) demonstrated that variation in LES traits is associated with plant height across species and identified key drivers of leaf trait variation among individuals at a global scale. They further proposed that plant size and LES traits may be linked by evaluating the variance in plant functional traits relative to plant size. This relationship may be explained by the notion that leaf traits and plant size represent independent axes of ecological and evolutionary diversity (Ackerly 2004). Nevertheless, how plant size mediates leaf trait differentiation between evergreen and deciduous subcommunities within forest communities remains poorly understood.

Plant size, a core indicator of plant growth and development status, plays an irreplaceable role in community assembly and the maintenance of ecosystem functions (Iida and Swenson 2020). It is commonly expressed through structural characteristics such as biomass accumulation and canopy position, which collectively modulate the capacity for resource acquisition, transport, and utilization (Enquist et al. 2007). Previous studies have suggested that larger plants generally occupy higher canopy strata, thereby enhancing light interception and photosynthetic carbon gain, whereas smaller individuals experience stronger light limitation and allocate proportionally more resources to height growth and leaf expansion to escape shading (Laurans et al. 2014). Furthermore, DeMalach et al. (2016) emphasized that differences in plant size determine competitive status within communities and mediate both intraspecific and interspecific interactions, such as competition and facilitation, ultimately shaping community dynamics and stability. Tumber‐Dávila et al. (2022) further found that increases in plant size are commonly accompanied by expanded root systems, enhancing access to spatially heterogeneous soil water and nutrient pools. Despite increasing evidence that plant size constrains resource availability and allocation during growth (Dovrat et al. 2019), it remains ambiguous how plant size influences leaf functional trait variation during growth.

Plant functional traits refer to morphological, physiological, and phenological characteristics that influence plant performance and are fundamental to assessing ecological adaptability (Díaz et al. 2016). Leaf functional traits, in particular, directly reflect strategic trade‐offs among carbon gain, nutrient investment, and tissue longevity, thereby linking measurable trait variation to underlying physiological and ecological functions (Bachmann et al. 2025). Wright et al. (2004) proposed the LES, which conceptualizes a continuous spectrum of leaf investment strategies ranging from resource‐acquisitive to conservative. The LES provides a unified framework for assessing coordinated variation under fluctuating environments (Henn and Damschen 2021) and also reflects resource allocation strategies during plant development through trait plasticity in leaf morphology and macronutrients across growth stages, which are closely associated with individual plant size (Wang et al. 2024). As plants grow, increasing biophysical constraints are accompanied by systematic changes in plant traits, as demonstrated by biophysical and size‐dependent analyses (Niklas 2013). However, it remains unclear how plant functional traits change with plant size, particularly in degraded karst ecosystems containing evergreen and deciduous subcommunities.

Karst ecosystems, characterized by inhomogeneous barren soil layers, extensive fissure development, high rock exposure, severe soil erosion, and nutrient deficiency, present high habitat heterogeneity and have experienced varying degrees of disturbance and degradation that influence plant growth and function (Li et al. 2021). Generally, the typical vegetation in karst ecosystems of southwest China is evergreen‐deciduous broad‐leaved mixed forests (Zhang et al. 2021). Previous studies in karst ecosystems have largely focused on interspecific variation in leaf functional traits or on community‐level functional characteristics (Liu et al. 2022). However, how plant size strategically differentiates leaf traits between evergreen and deciduous subcommunities in degraded karst forests remains unclear at present. Generally, deciduous woody species rely on rapid resource acquisition, maximizing photosynthetic gain during a relatively short leaf lifespan, whereas evergreen woody species follow a conservative resource‐use strategy, characterized by longer leaf lifespans and greater structural investment that sustains prolonged resource acquisition, as suggested by Wright et al. (2004) and Hikosaka et al. (2021). Accordingly, we hypothesized that deciduous subcommunities would exhibit more resource‐acquisitive leaf traits, whereas evergreen subcommunities would exhibit more conservative traits (H1). Moreover, previous studies suggested that plant size may alter the ability to capture light and impose associated physiological constraints (Wang et al. 2025), which may systematically and differentially affect the expression of resource use strategies in leaves of evergreen and deciduous subcommunities. We therefore further tested the hypothesis that plant size contributes differentially to leaf functional trait variation between subcommunities with different leaf habits. Specifically, given that deciduous species rely on rapid resource acquisition and generally exhibit greater phenotypic plasticity during growth and development, we predicted that plant size would exert a stronger influence on leaf trait variation in deciduous than in evergreen subcommunities (H2). Here, we examined plant size‐dependent differentiation in leaf functional traits between evergreen and deciduous subcommunities in degraded karst forests, aiming to advance our understanding of community assembly mechanisms and vegetation adaptation.

## 2 Materials and Methods

### 2.1 Study Area

**FIGURE 1:**
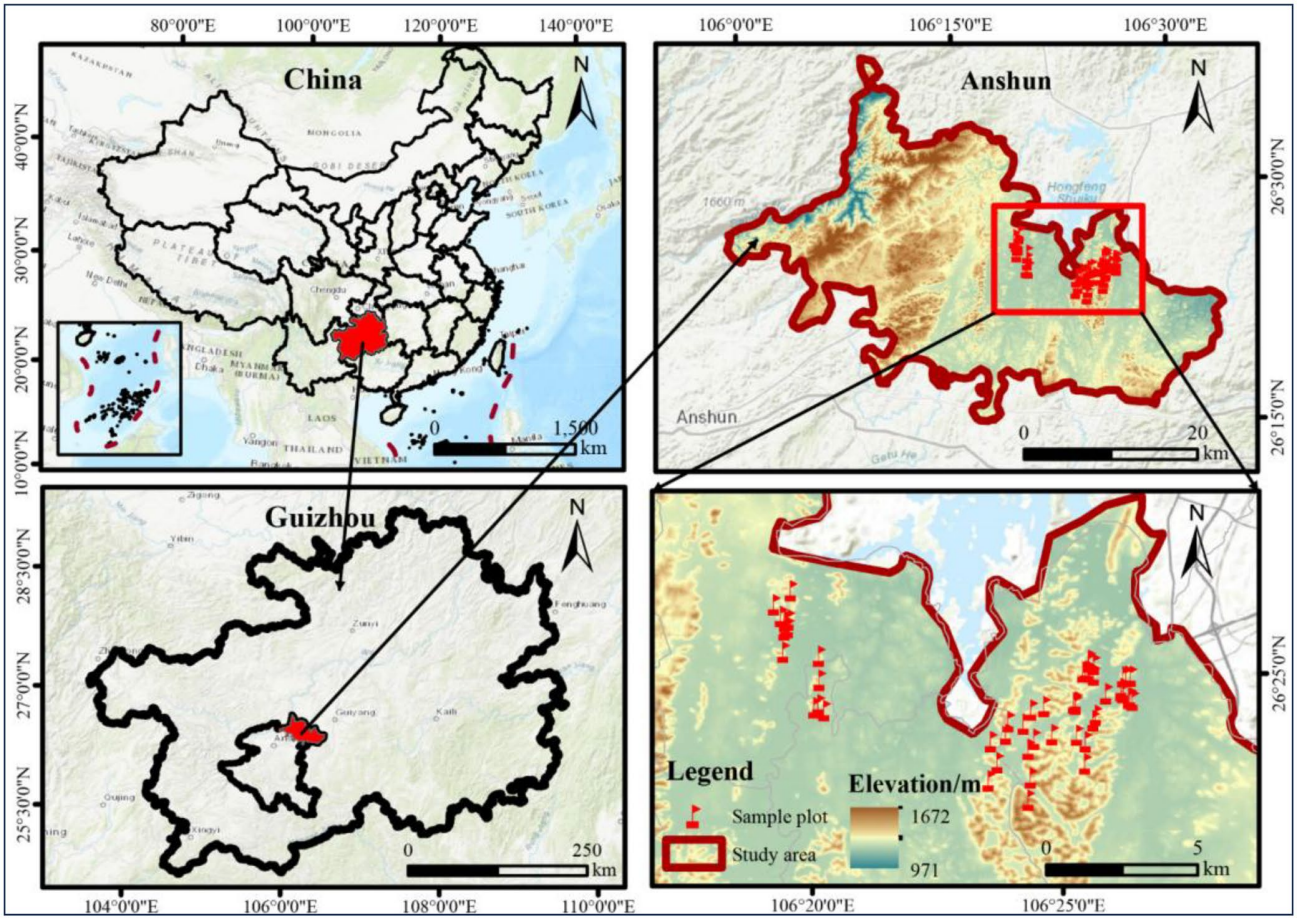
Geographic location and sampling site of the study areas.

This study was conducted in the typical degraded karst mountainous region of Pingba, Guizhou Province, southwest China, and extending from N 26°22′ to 26°28′ and from E 106°20′ to E 106°26′, at elevations ranging from 963 to 1646 m (Figure 1). The area exhibits climate characteristics of a subtropical humid monsoon, where annual temperature ranges from 14°C to 24°C and mean annual precipitation is approximately 1298 mm. These karst mountainous areas are underlain by typical limestone bedrock and are characterized by fragmented habitats, including extensive bedrock exposure and well‐developed rock fissures, as well as shallow, nutrient‐poor calcareous soils, resulting in high environmental heterogeneity and habitat degradation. The evergreen‐deciduous broad‐leaved mixed forest, a typical azonal vegetation under the subtropical climate of this region, is the dominant vegetation type and is generally regarded as the potential natural climax vegetation associated with karst topographic conditions. Representative evergreen species include *Ligustrum lucidum* , *Quercus phillyraeoides*, and *Myrsine Africana*, whereas the deciduous component is primarily composed of *Platycarya strobilacea, Coriaria nepalensis*, and *Rosa cymosa*. These woody species collectively form a modular community composed of evergreen and deciduous subcommunities differentiated by leaf habit.

### 2.2 Plant Community Survey and Sampling

A total of 53 forest plots (20 × 20 m) were randomly established for community surveys. Each plot was further divided by an adjacent grid system into four 10 × 10 m quadrats, resulting in a total of 212 subplots. The average distance between plots was 2018 m, ranging from a minimum of 50 m to a maximum of 4419 m. All plots were located on mid‐slope positions with similar aspects, with average values of slope gradient 15°, soil depth 25 cm, and rock exposure 55%. Initially, we surveyed plant communities in each plot by recording species names, individual counts, tree height, and diameter at breast height (DBH) for all stems with DBH ≥ 1 cm and heights exceeding 1.3 m, along with habitat information. Subsequently, branches from different orientations were collected from woody species to obtain mature, healthy leaves, which were placed in fresh bags for the measurement of leaf phenotypic traits and macronutrient traits of C, N, and P. We collected 1305 individual woody plants from 92 woody species across all plots, including 41 deciduous species (21 families, 37 genera) and 51 evergreen species (27 families, 37 genera), and leaf traits were measured. In total, 14,870 leaf trait observations were obtained, including 6067 measurements from evergreen species and 8803 measurements from deciduous species. Based on leaf habit, all surveyed woody species within each plot were classified into evergreen and deciduous subcommunities for subsequent analyses. In addition, soil samples were collected using a five‐point sampling method from the excavated soil profile in each plot, yielding 265 soil samples in total. Soil samples collected from each plot were transferred to the laboratory for the determination of soil physicochemical properties.

### 2.3 Evaluation of Leaf Traits and Soil Properties

Leaves collected in the field were measured for nine functional traits, including specific leaf area (SLA), leaf tissue density (LTD), leaf dry matter content (LDMC), leaf carbon content (LCC), leaf nitrogen content (LNC), leaf phosphorus content (LPC), and their stoichiometric ratios in this study. The leaf area (LA) was measured by analyzing scanned leaf images with WinRHIZO Pro software (LA2400), and leaf thickness (LT) was measured with a digital vernier caliper with a precision of 0.01 mm. The leaf fresh weight (LFW) was determined using a high‐precision electronic balance (1:10,000). After measurements of LA and LFW, samples were oven‐dried at 65°C for 48 h to constant weight. The leaf dry weight (LDW) was then recorded with the same electronic balance. Subsequently, the dried leaf samples were homogenized by a ball mill to determine leaf carbon, nitrogen, and phosphorus concentrations. LCC was quantified using the potassium dichromate method as described by Nelson and Sommers (1982), while LNC and LPC concentrations were determined using Kjeldahl digestion (BUCHI K‐360, Switzerland) and the molybdenum‐antimony colorimetric method (Murphy and Riley 1962), respectively. Stoichiometric ratios of leaf C, N, and P were calculated accordingly. Based on these measurements, the following leaf traits were calculated as follows:

(1) $$\mathrm{SLA}=\frac{\mathrm{LA}}{\mathrm{LDW}}$$

(2) $$\begin{array}{c} \text{LDMC}=\frac{\mathrm{LDW}}{\mathrm{LFW}} \end{array}$$

(3) $$\begin{array}{c} \mathrm{LTD}=\frac{\mathrm{LDW}}{\left(\mathrm{LA}\times \mathrm{LT}\right)} \end{array}$$

In addition, soil physicochemical properties were measured under sieving of 2 mm by an air‐dried treatment when removing plant roots and other impurities. Soil pH was measured using a pH meter with the potentiometric method; the soil organic carbon (SOC) was measured according to the potassium dichromate oxidation method as described by Bao (2000), and the total nitrogen (TN) and total phosphorus (TP) were analyzed using the consistent methods as those used for LNC and LPC in this study.

### 2.4 Community‐Weighted Traits and Habitat Heterogeneity

To characterize the ecological strategies of different subcommunities, the community‐weighted mean (CWM) of each leaf trait was calculated at the community level (Garnier et al. 2004; Muscarella et al. 2017). The CWM was computed as the abundance‐weighted mean of species trait values within each subcommunity, using the following formula:

(4) $$\begin{array}{c} \mathrm{CWM}=\sum_{i=1}^{n}\mathrm{RA}\times \text{trait} \end{array}$$

The CWM denotes the community‐weighted mean of a trait for each plot, *n* indicates the number of species per subcommunity, and RA*ᵢ* denotes the relative abundance of species *i* within the subcommunity, and trait is the measured value of the trait of species *i* obtained through measurement.

To qualify habitat heterogeneity, karst microhabitats within each plot were classified into six various components, including stone holes, grooves, rock surfaces, trenches, chinks, and soil surfaces according to Zhu (2003). In the field survey, a spline method was employed to establish profile lines at 1‐m intervals in both the vertical and horizontal directions of the plot (Lin and Liu 2018). The occurrence of each microhabitat type along the transects was recorded sequentially to determine their presence or absence. A heterogeneity index (Het) was then calculated to quantify horizontal spatial heterogeneity within each plot based on information entropy theory using the Shannon index, as implemented in the landscape diversity index (Shannon 1948). The index was calculated as follows:

(5) $$\begin{array}{c} \mathrm{Het}=-\sum_{i=1}^{m}\left(\mathrm{Pi}\right)\log\mathrm{Pi} \end{array}$$

where Het is the habitat heterogeneity index, *Pi* is the type of microhabitat, *i* is the probability of occurrence in the landscape, and *m* is the total number of microhabitat types.

### 2.5 Data Analysis

Leaf functional traits were quantified as plot‐level CWMs for evergreen and deciduous subcommunities, weighted by species relative abundance. Plant size was represented by the first principal component (PC1) derived from a PCA of plot‐level CWMs of DBH and tree height. Differences in CWM leaf traits between both subcommunities were tested using *t*‐tests in SPSS (28.0, IBM, Armonk, NY, USA), and relationships between plant size and CWM leaf traits were assessed using linear regression. Principal component analysis (PCA) was used to characterize multivariate relationships among leaf traits, soil properties, and habitat heterogeneity, while associations among variables were assessed using Spearman's rank correlation. Furthermore, to examine the effects of plant size, soil properties, and habitat quality with respect to leaf functional traits, nine traits were standardized, and the PC1, representing the main axis of trait variation, was extracted for each plot via PCA (Table S2). Loadings of leaf functional traits on the first two principal components for evergreen and deciduous subcommunities are presented (Table S3). Multiple linear regression models were fitted with leaf‐trait PC1 as the response variable, and the optimal set of predictors was identified based on AIC. Hierarchical partitioning was conducted in R using the “rdacca.hp” package to estimate the independent contributions of explanatory variables to variation in the leaf‐trait PC1. Standardized regression coefficients and variance contributions were visualized using forest plots and cumulative bar charts, respectively. Statistical computations and visualization were implemented in R version 4.4.1 (R Core Team, Vienna, Austria).

## 3 Results

### 3.1 Differences in Plant Size and Leaf Functional Traits Between Evergreen and Deciduous Subcommunities

**FIGURE 2:**
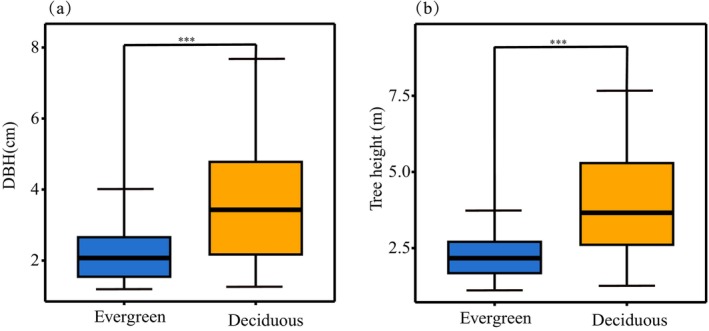
Differences in DBH (a) and tree height (b) of evergreen and deciduous subcommunities. ***p < 0.001.

Plant size differed significantly between subcommunities, with the deciduous subcommunity exhibiting greater DBH and height than the evergreen subcommunity in degraded karst forests (*p* < 0.001; Figure 2a,b). Furthermore, the deciduous subcommunity exhibited significantly greater community‐weighted mean values of SLA, LDMC, LNC, LPC, and leaf N/P ratio compared with the evergreen subcommunity. However, the evergreen subcommunity exhibited higher community‐weighted mean values of LCC, C/N, and C/P (Table 1). Overall, these trait differences suggest that the deciduous subcommunity generally adopts a more resource‐acquisitive strategy than the evergreen subcommunity.

**TABLE 1:** Community‐weighted means (CWM) of leaf functional traits in evergreen and deciduous subcommunities of degraded Karst mixed forests.

Traits	Type	Mean ± SD	*t*	*p*
SLA (cm^2^/g)	Deciduous	136.27 ± 29.87	4.96	< 0.001***
	Evergreen	111.08 ± 21.7		
LDMC (g/g)	Deciduous	0.46 ± 0.03	2.26	< 0.05*
	Evergreen	0.45 ± 0.04		
LTD(g/cm^3^)	Deciduous	0.46 ± 0.12	−1.27	0.21
	Evergreen	0.48 ± 0.09		
LCC (mg/g)	Deciduous	429.01 ± 13.67	−4.22	< 0.001***
	Evergreen	442.19 ± 18.16		
LNC (mg/g)	Deciduous	17.20 ± 1.92	10.17	< 0.001***
	Evergreen	13.49 ± 1.84		
LPC (mg/g)	Deciduous	0.90 ± 0.23	3.75	< 0.001***
	Evergreen	0.76 ± 0.17		
C/N	Deciduous	27.23 ± 3.78	−9.96	< 0.001***
	Evergreen	34.97 ± 4.24		
C/P	Deciduous	552.19 ± 133.80	−3.82	< 0.001***
	Evergreen	660.76 ± 157.83		
N/P	Deciduous	21.25 ± 5.43	2.22	< 0.05*
	Evergreen	19.15 ± 4.25		

Abbreviations: LCC, leaf carbon content; LDMC, leaf dry matter content; LNC, leaf nitrogen content; LPC, leaf phosphorus content; and their stoichiometric ratio traits of C/N, C/P, and N/P; LTD, leaf tissue density; SLA, specific leaf area.

* *p* < 0.05.

** *p* < 0.01.

*** *p* < 0.001.

### 3.2 Differential Effects of Plant Size on Leaf Functional Traits Across Evergreen and Deciduous Subcommunities

**FIGURE 3:**
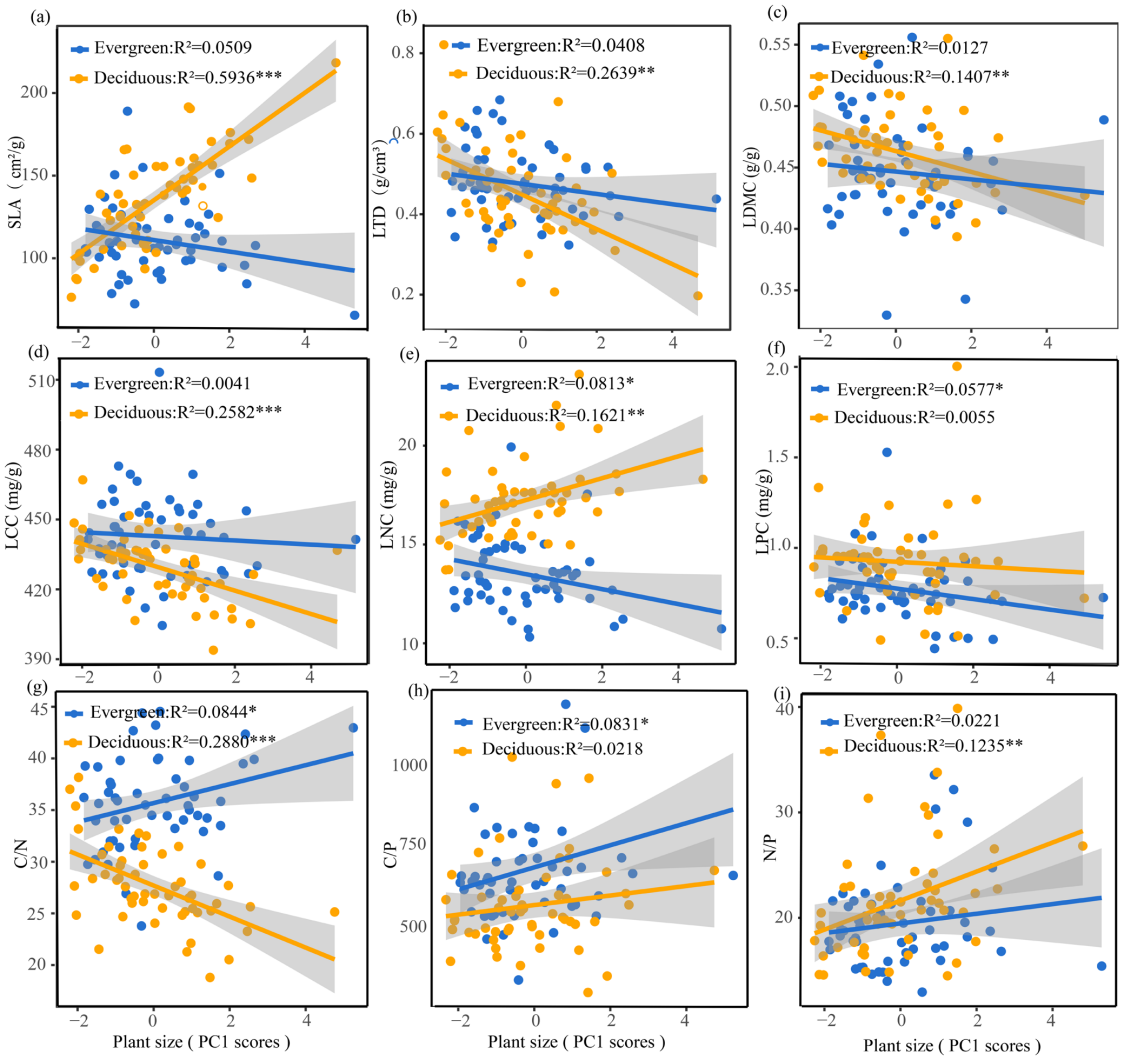
The correlations of plant size and leaf functional traits in evergreen and deciduous subcommunities. Plant size was determined using the scores by dimensionality reduction of DBH and tree height at PC1 axis. LCC, leaf carbon content; LDMC, leaf dry matter content; LNC, leaf nitrogen content; LPC, leaf phosphorus content and their stoichiometric ratio traits of C/N, C/P, and N/P; LTD, leaf tissue density; SLA, specific leaf area. *p < 0.05; **p < 0.01; ***p < 0.001.

PCA revealed a strong positive association between DBH and tree height in both subcommunities, with PC1 explaining 97.7% and 99.0% of the variance in the evergreen and deciduous subcommunities, respectively (Figure S1a,b). Therefore, PC1 scores were used as an integrated index of plant size in subsequent analyses (Table S1). Linear regression analyses revealed different relationships between plant size and leaf functional traits in the two subcommunities (Figure 3). In both subcommunities, LTD, LDMC, LCC, and LPC decreased with increasing plant size (Figure 3b–d,f), whereas leaf C/P and N/P ratios tended to increase (Figure 3h,i), showing either significant or non‐significant trends. However, several key traits responded differently between subcommunities. In the deciduous subcommunity, SLA and LNC increased significantly with plant size, whereas in the evergreen subcommunity, LNC decreased significantly, and SLA showed no significant relationship (Figure 3a,e). In addition, the leaf C/N ratio showed opposite trends between the two subcommunities (Figure 3g). Overall, plant size exerted different effects on leaf functional traits in evergreen and deciduous subcommunities, indicating divergent size‐dependent resource‐use strategies in degraded karst forests.

### 3.3 Trait Differentiations of Evergreen and Deciduous Subcommunities Linked to Soil Properties and Habitat Quality

**FIGURE 4:**
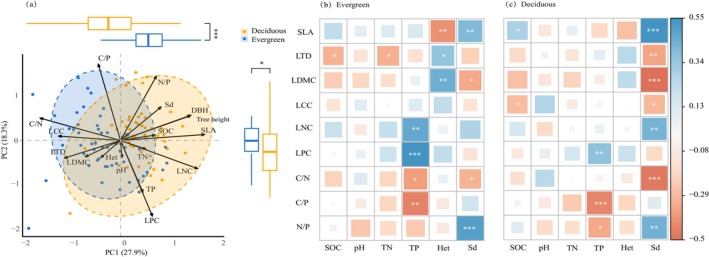
PCA (a) and correlation heatmap (b, c) for leaf functional trait deviation with plant size of DBH & height in association with habitat and soil properties. DBH, diameter at breast height; Het, heterogeneity index; SD, soil depth; SOC, soil organic carbon; TN, total nitrogen; TP, total phosphorus. For explanations of the abbreviations for leaf traits, see Figure 3. *p < 0.05; **p < 0.01; ***p < 0.001.

PCA based on leaf functional traits clearly differentiated evergreen and deciduous subcommunities along the PC1 axis (Figure 4a). PC1 and PC2 explained variations of 27.9% and 18.3%, respectively. The evergreen subcommunity was positioned primarily along the –PC1 axis, associated with high LCC and C/N, whereas the deciduous subcommunity was distributed along the +PC1 axis, associated with high SLA and LNC (Figure 4a). DBH and tree height were positively associated with the distribution of the deciduous subcommunity and negatively associated with the evergreen subcommunity. In the evergreen subcommunity, soil depth (Sd) was positively correlated with SLA and N/P but negatively related to LDMC and C/N. Habitat heterogeneity (Het) increased with LTD and LDMC but decreased with SLA, whereas TP decreased as C/N and C/P increased but increased with higher LNC and LPC (Figure 4b). In contrast, in the deciduous subcommunity, Sd demonstrated positive relationships with SLA, LNC, and N/P and negative relationships with LTD, LDMC, LCC, and C/N. Additionally, increasing TP was associated with lower C/P and N/P but higher LPC, whereas deciduous leaf traits showed no significant relationships with Het (Figure 4c). Overall, evergreen and deciduous subcommunities exhibited trait differentiation, with different associations with plant size, soil properties, and habitat quality.

### 3.4 Key Drivers of Leaf Trait Variation Associated With Plant Size, Soil Properties, and Habitat Quality

**FIGURE 5:**
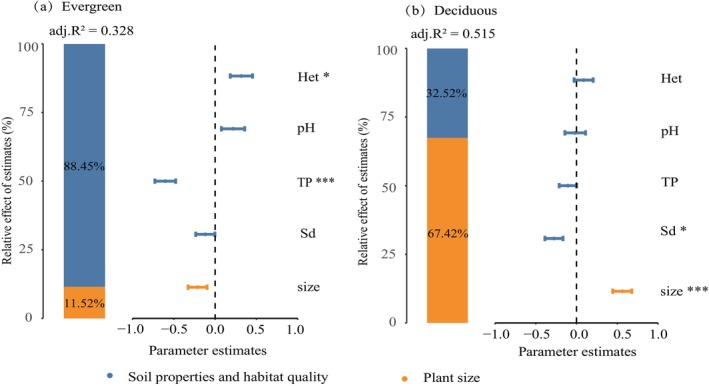
Relative effects of explanatory variables on leaf functional traits of evergreen (a) and deciduous (b) subcommunities. Bars indicate the relative importance of each driver, quantified as the proportion of explained variance. Error bars indicate 95% confidence intervals. *p < 0.05; ***p < 0.001.

Hierarchical partitioning analysis revealed contrasting drivers of leaf trait variation between evergreen and deciduous subcommunities (Table S2; Figure 5). The PC1 explained 40.9% and 40.6% of total trait variation in both subcommunities, respectively (Figure S2). The model explained a greater proportion of variation in the deciduous subcommunity (Adj.*R* ^2^ = 0.515) than in the evergreen subcommunity (Adj.*R* ^2^ = 0.328). In the evergreen subcommunity, trait variation was mainly associated with habitat quality and soil properties, with habitat heterogeneity exerting a positive effect, and TP showed a pronounced negative effect (Figure 5a, Table 2). In contrast, plant size had a positive effect on leaf trait variation in the deciduous subcommunity, whereas soil depth showed a negative effect. (Figure 5b, Table 2). Overall, habitat heterogeneity and soil phosphorus exerted the strongest influence on leaf traits in the evergreen subcommunity, whereas plant size was the dominant factor in the deciduous subcommunity.

**TABLE 2:** Multiple linear regression analysis results of the impact of soil properties and habitat heterogeneity on leaf functional traits.

Response variations	Variations	Parameter estimates	Standardard error	*T*‐value	*p*
Evergreen LFTs	Het	0.32	0.14	2.36	*
	pH	0.22	0.14	1.54	0.13
	Sd	−0.10	0.12	−0.90	0.37
	TP	−0.59	0.13	−4.69	***
	Size	−0.21	0.12	−1.84	0.07
Deciduous LFTs	Het	0.09	0.12	0.75	0.46
	pH	−0.02	0.13	−0.12	0.90
	Sd	−0.28	0.11	−2.50	*
	TP	−0.11	0.11	−0.99	0.33
	Size	0.56	0.12	4.82	***

Abbreviations: Het, heterogeneity index; LFTs, leaf functional traits; Sd, soil depth; TP, total phosphorus.

* *p* < 0.05.

** *p* < 0.01.

*** *p* < 0.001.

## 4 Discussion

### 4.1 Trait Differences and Strategy Differentiations of Evergreen and Deciduous Subcommunities

Deciduous species generally prioritize resource allocation to growth and light capture (Zhang et al. 2021), a strategy consistent with our observation that the deciduous subcommunity exhibited greater DBH and tree height than the evergreen subcommunity (Figure 2a,b). This suggests that the deciduous subcommunity in degraded karst habitats adopts a resource acquisition strategy. Similarly, the deciduous subcommunity showed greater SLA, LNC, and LPC (Table 1), further supporting a resource acquisition strategy. Within the leaf economics spectrum, high SLA, LNC, and LPC are associated with higher photosynthetic capacity and shorter leaf lifespan, as supported by Wright et al. (2004). These acquisitive traits generally facilitate rapid resource uptake and turnover, accelerate litter decomposition, and enhance nutrient cycling under seasonal resource fluctuation (Hikosaka et al. 2021). Although LDMC was also significantly greater in the deciduous subcommunity, trait coordination in degraded karst habitats may not fully conform to the conventional leaf economics spectrum trade‐off pattern because plants in these environments often experience simultaneous pressures from shallow soils, drought stress, and nutrient limitation. Previous studies have shown that stressful karst habitats can promote enhanced structural investment and conservative functional traits in plants (Fu et al. 2019). Under such conditions, some structural traits (e.g., LDMC) may increase to enhance stress tolerance while acquisitive nutrient‐use characteristics are still maintained. Therefore, we suggest that the higher LDMC observed in the deciduous subcommunity may reflect structural adaptation to karst stress rather than a purely conservative resource‐use strategy.

In contrast, the evergreen subcommunity showed greater LCC, C/N, and C/P (Table 1), consistent with a resource conservation strategy that prioritizes leaf structural defense and carbon storage, which in turn promotes leaf longevity and nutrient‐use efficiency (Qin et al. 2024). Furthermore, the evergreen subcommunity exhibited slightly higher LTD than the deciduous subcommunity, indicating stronger structural resistance (Krishna and Chandra Garkoti 2022). This aligns with the finding that evergreen woody plants strengthen their leaves via thicker cell walls and cuticles, using structural carbon compounds such as lignin and cellulose to improve toughness and drought tolerance (Yusuke et al. 2011). In addition, the high‐carbon structure of leaves also slows down litter decomposition, forming a cycle of high carbon input and slow turnover, matching the observations of Freschet et al. (2012). Therefore, these results support H1 that evergreen and deciduous subcommunities differ in leaf traits and resource‐use strategies. Additionally, both subcommunities exhibited leaf N/P above 16, reflecting that plant growth in this karst forest ecosystem is primarily limited by phosphorus availability (Wang et al. 2024). As proposed by Yan et al. (2017), the leaf N/P ratios are commonly used to assess the form of nutrient limitation in plants, with values below 14 indicating nitrogen limitation, values between 14 and 16 suggesting co‐limitation by nitrogen and phosphorus, and values above 16 reflecting phosphorus limitation. Overall, the deciduous subcommunity generally exhibited larger plant size and higher SLA, LNC, LPC, and N/P, indicating a resource acquisition strategy, whereas LDMC was also significantly greater, possibly reflecting structural adaptation to karst stress. In contrast, the evergreen subcommunity exhibited greater LCC, C/N, and C/P, presenting a conservative resource‐use strategy.

### 4.2 Plant Size‐Dependent Trait Differentiation in Evergreen and Deciduous Subcommunities

Our results indicated that plant size mediates the trade‐off between structural defense and resource acquisition in leaf traits. In the deciduous subcommunity, SLA, LNC, and leaf N/P ratio increased significantly, whereas the leaf C/N ratio decreased with increasing plant size (Figure 3a,e,g), suggesting that larger plant size promotes higher photosynthetic capacity and faster nitrogen turnover, consistent with Qi et al. (2024). Furthermore, Ishii et al. (2013) found that enhanced resource acquisition associated with increasing plant size is mainly driven by greater demands for light capture during canopy competition, accompanied by allocation toward photosynthetic tissues rather than structural defenses, which may explain the greater resource‐use efficiency associated with larger plant size in the deciduous subcommunity. These patterns suggest that increasing plant size promotes a shift toward a more resource‐acquisitive strategy in the deciduous subcommunity, characterized by enhanced resource capture and rapid nutrient turnover. In contrast, in the evergreen subcommunity, LNC decreased significantly, whereas leaf C/N and C/P ratios increased with plant size, and SLA showed a non‐significant declining trend (Figure 3a,e,g), suggesting slower nutrient turnover and greater investment in leaf structural resistance as plants grow larger, as supported by Hikosaka et al. (2025). Additionally, previous studies found that evergreen woody species with longer leaf lifespans and higher construction costs tend to stabilize leaf structural and functional traits during growth (Li et al. 2025), which helps maintain long‐term carbon gain and nutrient‐use efficiency. This conservative pattern may reflect the need to maintain leaf longevity and nutrient retention throughout ontogeny, thereby ensuring sustained returns from leaves with high construction costs.

Furthermore, our results showed that with increasing plant size, the deciduous subcommunity exhibited significant decreases in LTD, LDMC, and LCC, whereas the evergreen subcommunity showed no significant declines in these traits, despite a decrease in LPC (Figure 3b–d,f). The size‐dependent responses indicate that the deciduous subcommunity progressively shifts toward a more resource‐acquisitive strategy as plant size increases. Greater light competition in larger individuals may promote allocation to photosynthetic tissues and carbon assimilation rather than to structurally costly leaf tissues (Freschet et al. 2010), resulting in lower LTD, LDMC, and LCC. In contrast, the evergreen subcommunity continues to maintain a relatively conservative strategy with increasing plant size. Their longer leaf lifespan and higher construction costs necessitate sustained structural investment to maximize carbon payback and maintain stress resistance over extended periods (Bai et al. 2015; Reich and Cornelissen 2014). Overall, our results indicate that plant size promotes strategic differentiation in leaf functional traits between evergreen and deciduous subcommunities by mediating trade‐offs between structural investment and nutrient acquisition. The evergreen subcommunity favors slower and more conservative resource use, while the deciduous subcommunity shifts toward rapid resource acquisition with increasing plant size.

### 4.3 Subcommunity Strategy Mechanisms of Trait Differentiation

PCA revealed clear differentiation between evergreen and deciduous subcommunities along the primary trait axis (Figure 4a), representing a continuum from conservative to acquisitive leaf economic strategies consistent with the leaf economics spectrum (Díaz et al. 2016; Wright et al. 2004). Importantly, our results showed that the determinants of leaf traits differed markedly between subcommunities, suggesting that subcommunities with different leaf habits rely on different mechanisms to cope with environmental constraints in degraded karst forests.

Leaf trait variation in the deciduous subcommunity was primarily driven by plant size. This pattern suggests that plant growth is accompanied by a progressive shift in the trade‐off between structural defense and resource acquisition. Studies have shown that as plant size increases, both light availability and carbon accumulation tend to increase, enhancing overall resource acquisition capacity and allowing the deciduous subcommunity to adjust leaf functional traits toward higher photosynthetic returns and nutrient content while reducing structural investment (Li et al. 2022). This size‐dependent enhancement of resource acquisition is particularly important for the deciduous subcommunity, as they rely on short‐lived leaves and rapid resource uptake to sustain growth and competitive ability (Edwards et al. 2014; Qi et al. 2024). In addition, larger deciduous individuals may accumulate greater resource reserves during the leaf‐on period, which can be mobilized and used to sustain metabolic demands and support survival during the leaf‐off period (Luo et al. 2024). As a result, the deciduous subcommunity exhibits a more progressive transition toward acquisitive leaf traits as plant size increases. This highlights why plant size, as a key determinant of resource acquisition, emerges as a primary driver of trait variation in this subcommunity.

Conversely, leaf traits in the evergreen subcommunity were mainly influenced by soil phosphorus and habitat heterogeneity (Figure 5a). The evergreen subcommunity generally maintains long‐lived leaves with high structural investment, resulting in slower nutrient turnover and stronger dependence on sustained nutrient supply (Krishna and Chandra Garkoti 2022). In karst ecosystems, phosphorus is a limiting resource despite relatively high total phosphorus concentrations because alkaline, calcium‐rich soil reduces phosphorus availability through precipitation and adsorption (Patel and Goswami 2020). Under such conditions, evergreen subcommunities may be particularly sensitive to variation in phosphorus availability because maintaining long‐lived leaves requires continuous nutrient support for metabolic maintenance and structural integrity (Chen et al. 2023). Habitat heterogeneity further increases spatial variation in soil depth, moisture, and nutrient availability, which may increase environmental filtering and promote conservative resource‐use strategies characterized by greater stress tolerance (Ma et al. 2025; Madhubhani et al. 2025). In our study, evergreen subcommunities exhibited lower SLA and higher LDMC in response to increased habitat heterogeneity (Figure 4b), suggesting a shift toward a more conservative strategy that prioritizes leaf structural investment and longevity over rapid resource acquisition.

These contrasting responses are consistent with the fast‐slow plant economics framework (Wright et al. 2004). The deciduous subcommunity lies toward the acquisitive side of the resource‐use continuum, characterized by rapid resource capture, high nutrient turnover, and flexible adjustment of leaf traits in response to increasing plant size. The evergreen subcommunity, by contrast, occupies the conservative end of the spectrum, maintaining durable leaves and stable trait syndromes that prioritize long‐term carbon return and nutrient retention over rapid resource acquisition (Hikosaka et al. 2025). Our findings therefore suggest that plant size and environmental filtering operate differently between evergreen and deciduous subcommunities, reinforcing divergence in resource‐use strategies between evergreen and deciduous subcommunities. From a community assembly perspective, our results suggest that environmental filtering and plant size‐mediated trait differentiation jointly contribute to species persistence within evergreen and deciduous subcommunities in degraded karst forests. Environmental constraints associated with soil phosphorus availability and habitat heterogeneity play a major role in shaping leaf trait variation in evergreen subcommunities, whereas increasing plant size promotes acquisitive trait adjustments in deciduous subcommunities. However, the observational nature of this study precludes direct causal inference, and future experimental studies are needed to verify the underlying mechanisms.

## 5 Conclusions

This study demonstrated that plant size plays a pivotal role in shaping leaf trait variation and ecological strategies in evergreen and deciduous subcommunities in degraded karst forests. Leaf traits differed significantly between the two subcommunities, with the deciduous subcommunity exhibiting greater tree DBH, height, SLA, LDMC, LNC, LPC, and N/P ratios, but lower LCC, C/N, and C/P ratios compared with the evergreen subcommunity, indicating clear differentiation in resource‐use strategies among modular subcommunities in karst forests. In addition, leaf traits showed significant relationships with plant size, particularly for SLA, LNC, and C/N in both subcommunities, suggesting a size‐dependent pattern of trait differentiation and ecological strategies. Moreover, the relative effects indicated that evergreen leaf traits were mainly influenced by soil total phosphorus and habitat heterogeneity, whereas deciduous leaf traits were primarily driven by plant size. Overall, we suggest that modular subcommunities exhibit distinct resource‐use strategies, with an acquisitive strategy in the deciduous subcommunity and a conservative strategy in the evergreen subcommunity, closely associated with plant size in degraded karst forests. This study contributes to our understanding of trait‐based community assembly by demonstrating that plant size‐dependent modulation of the leaf economics spectrum (LES) adds a new dimension to community assembly. Our findings highlight that plant size should be explicitly considered as a key axis of functional variation, which is important for elucidating the mechanisms underlying community assembly processes and ecosystem functioning. These findings also provide practical implications for species selection, stand development, and adaptive management in the ecological restoration of degraded karst ecosystems.

## Author Contributions

**Mingjuan Yu:** data curation (equal), formal analysis (equal), investigation (equal), writing – original draft (equal). **Min Jiao:** investigation (equal), writing – review and editing (equal). **Xihong Yang:** investigation (equal), writing – review and editing (equal). **Xiaodong Li:** writing – review and editing (equal). **Yuejun He:** conceptualization (equal), formal analysis (equal), funding acquisition (equal), supervision (equal), writing – review and editing (equal).

## Funding

This work was supported by Central Funding for National Nature Reserves (Grant CFNR‐2025‐XS‐R01), National Key Research and Development Project of China (Grant 2025YFC2609500), National Natural Science Foundation of China (Grant NSFC:32260268), and Guizhou Provincial Basic Research Foundation (Grant ZD [2025] 072).

## Conflicts of Interest

The authors declare no conflicts of interest.

## Supporting information


**Figure S1:** PCA for DBH and tree height in evergreen and deciduous subcommunities.
**Figure S2:** PCA for leaf functional traits in evergreen and deciduous subcommunities.
**Table S1:** Principal component scores (PC1 and PC2) of diameter at breast height (DBH) and tree height for evergreen and deciduous subcommunities.
**Table S2:** Principal component scores (PC1 and PC2) of leaf traits for evergreen and deciduous subcommunities.
**Table S3:** Loadings of leaf functional traits on the first two principal components (PC1 and PC2) in evergreen and deciduous subcommunities.

## Data Availability

The dataset used in this study is available at the following: https://zenodo.org/records/18506311.
