# Supplementary material for: Leaf Trait Differentiations Depend on Plant Size in a Modular Assembled Community of Evergreen and Deciduous Trees From a Degraded Karst Forest Ecosystem
Source: Ecol Evol. 2026 Jul 8;16(7):e73996. doi: 10.1002/ece3.73996 (PMC13343730; doi:10.1002/ece3.73996)
Supplement: Supplementary file 1 — Figure S1: PCA for DBH and tree height in evergreen and deciduous subcommunities. Figure S2: PCA for leaf functional traits in evergreen and deciduous subcommunities. Table S1: Principal component scores (PC1 and PC2) of diameter at breast height (DBH) and tree height for evergreen and deciduous subcommunities. Table S2: Principal component scores (PC1 and PC2) of leaf traits for evergreen and deciduous subcommunities. Table S3: Loadings of leaf functional traits on the first two principal components (PC1 and PC2) in evergreen and deciduous subcommunities. [file ECE3-16-e73996-s001.docx]

**Supplement Materials**

**Figures**

**

**

**Fig. S1** PCA for DBH and tree height in evergreen and deciduous subcommunities


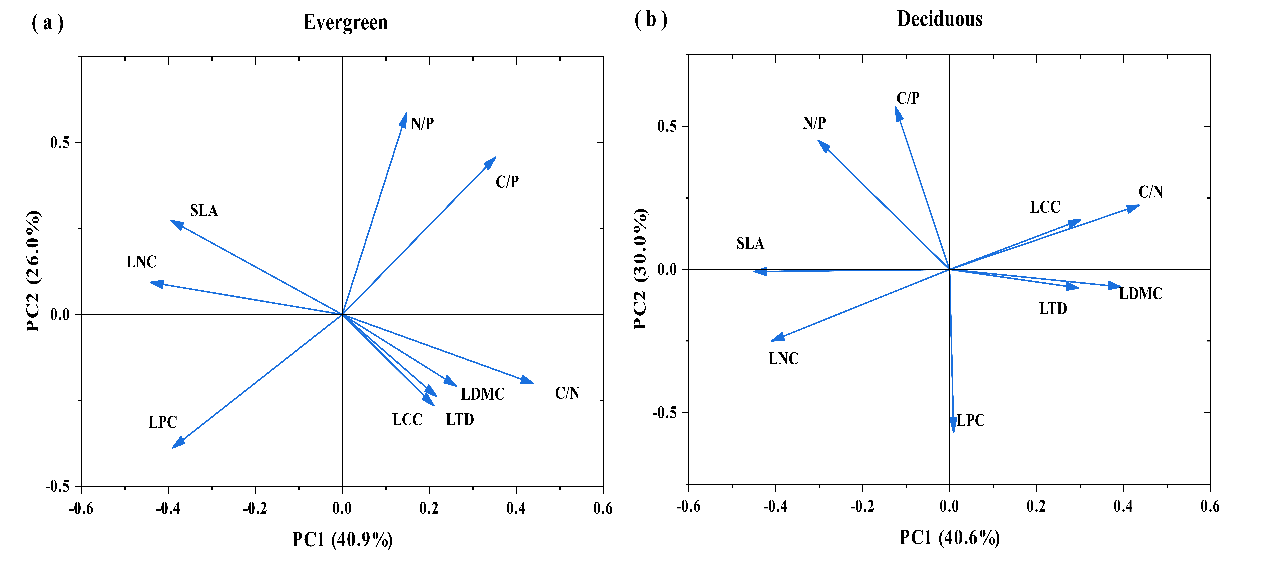


**Fig. S2** PCA for leaf functional traits in evergreen and deciduous subcommunities

**Tables**

| **Table S1** Principal component scores (PC1 and PC2) of diameter at breast height (DBH) and tree height for evergreen and deciduous subcommunities | | | | |
| --- | --- | --- | --- | --- |
| **Plot-ID** | **Evergreen** | | **Deciduous** | |
|  | **PC1 scores** | **PC2 scores** | **PC1 scores** | **PC2 scores** |
| 1 | -1.2956 | 0.0393 | -0.9102 | -0.0597 |
| 2 | 0.7466 | -0.1011 | -0.0137 | -0.1121 |
| 3 | -0.5420 | 0.2352 | -4.7567 | 0.4124 |
| 4 | -0.5733 | -0.0661 | 0.2883 | 0.0438 |
| 5 | -0.9261 | 0.3143 | 0.5334 | -0.0067 |
| 6 | 1.0473 | 0.1843 | 1.3803 | 0.1139 |
| 7 | -1.3940 | 0.2755 | -0.7361 | 0.0102 |
| 8 | -0.0857 | -0.1371 | 0.5056 | 0.1172 |
| 9 | 1.0296 | -0.0669 | 0.8986 | 0.1778 |
| 10 | -5.2497 | -0.8280 | -1.9542 | -0.1868 |
| 11 | 1.5644 | -0.0346 | -1.9697 | 0.2880 |
| 12 | -1.7531 | 0.1986 | -0.9846 | -0.2625 |
| 13 | 0.4999 | -0.0884 | -2.4530 | 0.0682 |
| 14 | -0.8903 | 0.0759 | -1.4945 | -0.0318 |
| 15 | 1.4912 | 0.0058 | -0.9650 | 0.0752 |
| 16 | -0.9245 | -0.0914 | -0.6414 | -0.1794 |
| 17 | -2.4027 | -0.0915 | -0.3819 | -0.3403 |
| 18 | 1.3507 | -0.1841 | -2.5340 | -0.1448 |
| 19 | 1.5469 | -0.1182 | -0.7045 | -0.1934 |
| 20 | 0.9134 | -0.1982 | -1.2322 | -0.0983 |
| 21 | 1.2073 | -0.2580 | -0.8117 | -0.1708 |
| 22 | 0.9722 | 0.0544 | 1.6280 | 0.0371 |
| 23 | 1.1623 | -0.1170 | 2.0577 | -0.0045 |
| 24 | 1.1368 | -0.0171 | 0.2117 | -0.0426 |
| 25 | -0.3411 | -0.0371 | -0.5495 | -0.0966 |
| 26 | 0.3207 | -0.0137 | 0.2473 | 0.0168 |
| 27 | -0.1133 | 0.2186 | 0.6117 | 0.1413 |
| 28 | -0.0365 | 0.1230 | 0.2870 | 0.1459 |
| 29 | 1.1635 | 0.1582 | 2.2279 | 0.1769 |
| 30 | 0.5559 | -0.0662 | 2.1081 | -0.0131 |
| 31 | 1.0839 | -0.0267 | 0.4054 | 0.1114 |
| 32 | 1.8248 | 0.0883 | 2.0767 | 0.1775 |
| 33 | 0.0660 | -0.0664 | -0.2156 | -0.0455 |
| 34 | 0.3394 | 0.0702 | -1.4691 | 0.2081 |
| 35 | -0.3142 | -0.5223 | 0.9323 | -0.1803 |
| 36 | -1.6861 | 0.6740 | 1.4618 | 0.1025 |
| 37 | -1.7490 | 0.1640 | -0.0077 | 0.1400 |
| 38 | 1.2243 | 0.0570 | -1.6461 | 0.0661 |
| 39 | -1.1428 | 0.1312 | 0.8306 | -0.0852 |
| 40 | 1.4680 | -0.0528 | 0.3279 | 0.1639 |
| 41 | -2.6325 | 0.4388 | 0.8561 | -0.0280 |
| 42 | 0.4508 | -0.1141 | -0.2089 | -0.1231 |
| 43 | 0.5133 | -0.1334 | 0.9901 | -0.0817 |
| 44 | 0.2366 | 0.0459 | 0.9367 | -0.1519 |
| 45 | 0.9007 | 0.0987 | 2.0038 | 0.0964 |
| 46 | -0.1510 | -0.0214 | 0.5856 | -0.0207 |
| 47 | -0.7935 | -0.0564 | 1.2974 | 0.0907 |
| 48 | 1.7561 | -0.0937 | -1.1390 | -0.1060 |
| 49 | 1.8568 | -0.0001 | 0.7642 | -0.0372 |
| 50 | -0.7689 | 0.1706 | -1.0108 | 0.0248 |
| 51 | -1.0516 | 0.0694 | 1.1989 | -0.0878 |
| 52 | -2.3448 | -0.1571 | -0.8618 | -0.0980 |
| 53 | 0.7333 | -0.1323 | 1.9987 | -0.0174 |

| **Table S2** Principal component scores (PC1 and PC2) of leaf traits for evergreen and deciduous subcommunities | | | | |
| --- | --- | --- | --- | --- |
| **Plot-ID** | **Evergreen** | | **Deciduous** | |
|  | **PC1 scores** | **PC2 scores** | **PC1 scores** | **PC2 scores** |
| 1 | -0.9460 | -1.3771 | -2.2618 | 0.6394 |
| 2 | -4.0023 | -2.0908 | -1.7960 | -0.5457 |
| 3 | -0.6915 | -0.6895 | -3.0476 | 1.3287 |
| 4 | -0.8223 | 2.1904 | 0.2141 | -1.9873 |
| 5 | 0.7894 | 0.7415 | 0.3274 | 0.0420 |
| 6 | -0.1666 | 0.4613 | 0.7184 | 2.0756 |
| 7 | 1.2363 | -4.0019 | -2.0114 | 1.6209 |
| 8 | 1.4942 | 0.0181 | -1.0912 | 4.5213 |
| 9 | -0.6621 | 1.4173 | 1.9199 | -1.4379 |
| 10 | 2.3100 | 1.6426 | -1.8220 | 0.4616 |
| 11 | -2.2963 | 0.1077 | -2.6724 | -3.1284 |
| 12 | 2.3299 | -2.6829 | -0.5043 | 1.8219 |
| 13 | 0.2901 | -1.0274 | -2.4298 | 0.2729 |
| 14 | 2.2667 | -4.1408 | -3.0815 | 3.5675 |
| 15 | 2.1063 | -0.4275 | -2.8687 | 1.5254 |
| 16 | 1.9382 | -2.9192 | -0.9794 | 3.6108 |
| 17 | 3.1625 | -1.8368 | -0.8897 | 0.9570 |
| 18 | -1.2733 | -0.5271 | -0.8613 | -0.0750 |
| 19 | -0.0536 | 0.3361 | 0.1437 | 0.4329 |
| 20 | -1.1428 | -1.5085 | 2.1741 | -1.8835 |
| 21 | 1.2288 | -0.0345 | -0.5052 | -0.2541 |
| 22 | 0.0586 | 1.7131 | 1.8725 | -0.6846 |
| 23 | -1.5823 | 2.3053 | 1.3899 | -0.9958 |
| 24 | 0.1147 | 0.2432 | 1.4622 | 0.3641 |
| 25 | -0.9435 | -0.0169 | -0.1306 | -0.0585 |
| 26 | 1.4415 | 1.0460 | 1.1852 | 0.2898 |
| 27 | 0.6237 | 0.2607 | -0.0535 | -0.7597 |
| 28 | 2.6230 | 1.8562 | 2.4029 | 0.1685 |
| 29 | 1.2970 | 1.2696 | 3.9132 | 0.7067 |
| 30 | 2.7730 | 3.2122 | 1.7925 | -2.2649 |
| 31 | -1.4885 | -0.7683 | 1.5005 | 0.8367 |
| 32 | 1.0522 | 0.7824 | 3.7458 | 1.4606 |
| 33 | 0.2411 | -1.0163 | -0.2092 | -1.4207 |
| 34 | -7.5334 | 0.6435 | -4.0650 | -5.8132 |
| 35 | 1.0978 | 1.2557 | 1.8617 | -1.6380 |
| 36 | -4.1185 | -1.2644 | -1.9156 | -1.2005 |
| 37 | -0.5000 | 0.5528 | 1.1855 | -0.7139 |
| 38 | -1.5022 | 0.8497 | 0.2501 | -0.6074 |
| 39 | -0.8241 | 0.4672 | -2.1119 | 0.9980 |
| 40 | 0.5581 | 0.2612 | 1.2344 | 1.0915 |
| 41 | 0.5114 | 0.0285 | 0.6927 | 0.3394 |
| 42 | -1.3118 | 0.6565 | 0.6437 | -0.3178 |
| 43 | -1.0685 | -0.5744 | 0.3275 | -0.8609 |
| 44 | -0.4871 | -0.7668 | 1.5981 | -0.1479 |
| 45 | 2.0026 | 0.1222 | 2.6458 | 0.0156 |
| 46 | 2.0818 | 0.4463 | -0.4419 | -0.6429 |
| 47 | 0.7919 | -0.5256 | 0.5204 | 0.1663 |
| 48 | -2.4315 | -1.5605 | -1.2909 | -0.5443 |
| 49 | -0.1240 | 0.0440 | -0.5611 | 0.4020 |
| 50 | -0.8926 | -0.0622 | -0.3846 | -0.5780 |
| 51 | -0.5892 | 1.1447 | 1.4633 | -0.3672 |
| 52 | 1.1913 | 0.5116 | -3.1160 | -1.3564 |
| 53 | -0.1580 | 3.2316 | 3.9169 | 0.5675 |

| **Table S3** Loadings of leaf functional traits on the first two principal components (PC1 and PC2) in evergreen and deciduous subcommunities | | | | |
| --- | --- | --- | --- | --- |
| **Traits** | **Evergreen** | | **Deciduous** | |
|  | **PC1 scores** | **PC2 scores** | **PC1 scores** | **PC2 scores** |
| SLA | -0.3944 | -0.2738 | -0.4495 | -0.0073 |
| LDMC | 0.2618 | 0.2087 | 0.3931 | -0.0607 |
| LTD | 0.2165 | 0.2389 | 0.2968 | -0.0648 |
| LCC | 0.2102 | 0.2649 | 0.3016 | 0.1731 |
| LNC | -0.4408 | -0.0936 | -0.4093 | -0.2498 |
| LPC | -0.3904 | 0.3883 | 0.0091 | -0.5687 |
| C/N | 0.4389 | 0.2005 | 0.4357 | 0.2243 |
| C/P | 0.3519 | -0.4578 | -0.1248 | 0.5680 |
| N/P | 0.1474 | -0.5873 | -0.3023 | 0.4508 |

**NOTE:** SLA = specific leaf area; LTD = leaf tissue density; LDMC = leaf dry matter content; LCC =leaf carbon content; LNC =leaf nitrogen content; LPC =leaf phosphorus content and their stoichiometric ratio traits of C/N, C/P, and N/P.
